# Risk Stratification in Pulmonary Embolism: The Expanding Role of Biomarkers

**DOI:** 10.3390/biomedicines14051046

**Published:** 2026-05-04

**Authors:** Cyrus Moini, Piseth Lay, Sebastien Jochmans, Fidele Azandjo, Nassima El Karroumi, Anne-Laure Bouilland, El Mahdi Hafiani

**Affiliations:** 1 Cardiology Department, Groupe Hospitalier Sud-Ile de France, 270 Avenue Marc Jacquet, 77000 Melun, France; cyrus.moini@ghsif.fr (C.M.); piseth.lay@ghsif.fr (P.L.); fidele.azandjo@ghsif.fr (F.A.); nassima.elkarroumi@ghsif.fr (N.E.K.); 2 Department of Intensive Care Medicine, Groupe Hospitalier Sud Ile de France, 270 Avenue Marc Jacquet, 77000 Melun, France; sebastien.jochmans@ghsif.fr (S.J.); annelaure.bouilland@ghsif.fr (A.-L.B.); 3 Department of Anesthesia and Perioperative Medecine, Private Hospital of Antony, 1 Rue Velpeau, 92160 Antony, France

**Keywords:** pulmonary embolism, risk stratification, prognosis, biomarkers, troponin, BNP/NT-proBNP, D-dimer, copeptin, heart-type fatty acid-binding protein (H-FABP), lactate, microRNAs, inflammatory biomarkers

## Abstract

Pulmonary embolism (PE) remains a frequent and potentially fatal condition, with early mortality largely driven by (RV) failure and hemodynamic collapse. Rapid and accurate prognostic assessment is therefore central to management. Current European Society of Cardiology (ESC) strategies rely first on hemodynamic status to identify high-risk patients requiring urgent reperfusion consideration, and then—when patients are normotensive—on a stepwise approach combining clinical risk scores, RV imaging, and circulating biomarkers. Clinical tools such as HESTIA and the Pulmonary Embolism Severity Index (PESI)/simplified PESI (sPESI) enable early identification of low-risk patients suitable for outpatient pathways and stratify 30-day mortality risk, but do not integrate biological data. Consequently, biomarkers have an expanding role in refining prognosis, particularly within the heterogeneous intermediate-risk group. This review provides a practical overview of established and emerging biomarkers for PE risk stratification. Conventional cardiac biomarkers—troponins and natriuretic peptides (BNP/NT-proBNP)—reflect RV myocardial injury and strain and, when combined with imaging evidence of RV dysfunction, allow discrimination between intermediate–low- and intermediate–high-risk PE, guiding monitoring intensity and escalation strategies. D-dimer, while essential in diagnostic algorithms because of its high negative predictive value, has only an adjunctive and indirect prognostic role. Beyond these markers, growing evidence supports additional biomarkers capturing complementary pathways: neurohormonal stress (copeptin), early myocardial injury (H-FABP), inflammation and hypoxia (GDF-15), tissue hypoperfusion (lactate), and molecular regulation (circulating microRNAs). Readily available inflammatory indices derived from blood counts (NLR, PLR, LMR), red cell distribution width, and hs-CRP may further contribute within multimarker models, although specificity and validation remain limitations. Future directions include multimodal and omics-driven biomarker profiling integrated with advanced imaging to enable more precise, dynamic, and personalized PE care, from acute risk prediction to long-term follow-up and prevention of chronic thromboembolic complications.

## 1. Introduction

Pulmonary embolism (PE) is a common and potentially fatal disease [1,2]. Its annual incidence in Europe is estimated at 60–80 cases per 100,000 inhabitants [3]. Despite advances in diagnosis and management, PE remains associated with substantial mortality, ranging from 3% to 12% within 30 days after diagnosis, and reaching up to 50% among patients classified as high risk for early adverse events [2,4].

The major challenge in PE management is the rapid and accurate assessment of prognosis. Since 2014, European guidelines for the diagnosis and management of acute PE have relied primarily on risk stratification based on hemodynamic status. Hemodynamic instability immediately identifies patients at high risk and mandates urgent treatment with unfractionated heparin and consideration of reperfusion therapies such as systemic thrombolysis or thrombectomy.

In the absence of hemodynamic instability, prognostic assessment relies on a stepwise, multimodal strategy combining clinical risk scores, imaging assessment of right ventricular (RV) dysfunction, and circulating cardiac biomarkers. Importantly, most widely used clinical risk scores, such as PESI/sPESI and HESTIA, do not incorporate biomarkers. However, some prognostic models, including the BOVA and FAST scores, partially integrate clinical variables, imaging findings, and/or biomarkers, although none are currently recommended as standalone tools in ESC risk-adapted management algorithms [5].

## 2. Clinical Risk Scores in Hemodynamically Stable Pulmonary Embolism

For hemodynamically stable patients, clinical risk is initially assessed using validated tools such as the HESTIA criteria, the Pulmonary Embolism Severity Index (PESI), or its simplified version (sPESI). Among these, the sPESI is the most widely used in clinical practice and forms the cornerstone of current European risk-adapted management algorithms [2].

### 2.1. HESTIA Criteria

The HESTIA criteria (Table 1) were developed to identify patients with acute PE who are eligible for early discharge and outpatient management. This tool is a binary checklist based exclusively on clinical, organizational, and social factors [6]. The presence of any criterion indicates that outpatient treatment is not appropriate. Notably, HESTIA does not estimate mortality risk and does not include imaging findings or biomarkers.

Interpretation:

- No HESTIA criteria present → outpatient treatment may be considered
- ≥1 criterion present → inpatient management recommended

HESTIA is a pragmatic tool that facilitates safe outpatient selection but does not provide prognostic information regarding short-term mortality [7].

The safety of outpatient management strategies based on the HESTIA criteria has been supported by randomized controlled trials and systematic reviews. In the VESTA trial, outpatient treatment guided by the HESTIA rule was shown to be safe, with low rates of recurrent venous thromboembolism, major bleeding, and mortality, whether or not N-terminal pro-brain natriuretic peptide (NT-proBNP) testing was added.

Furthermore, meta-analyses and Cochrane reviews have confirmed that carefully selected low-risk patients with acute PE can be safely managed in an outpatient setting using structured clinical decision rules. These findings support the use of HESTIA as part of an evidence-based ambulatory management strategy, potentially complemented by biomarkers to further enhance patient selection [8].

### 2.2. Simplified Pulmonary Embolism Severity Index (sPESI)

The sPESI (Table 2) is a validated prognostic score designed to estimate 30-day all-cause mortality in patients with acute PE. It is derived from the original PESI and includes six clinical variables, each contributing one point [9]. Biomarkers and imaging parameters are not included in the sPESI score.

Patients with an sPESI score of 0 are classified as low risk and may be considered for early discharge or outpatient treatment, provided that RV imaging and cardiac biomarkers are normal. Patients with sPESI ≥ 1 require further risk stratification using echocardiography or computed tomography to assess RV dysfunction, as well as measurement of cardiac biomarkers (e.g., troponin, BNP or NT-proBNP).

### 2.3. Comparison and Clinical Use

In clinical practice, HESTIA and sPESI are complementary rather than competing tools. HESTIA is particularly useful for safe outpatient selection, whereas sPESI forms the basis of prognostic stratification within the ESC risk-adapted management algorithm [6,9]. Table 3 provides a side-by-side comparison of the HESTIA and sPESI scores, outlining their differences in structure, purpose, and integration into clinical management strategies.

### 2.4. Place of Clinical Scores Within the ESC Risk Stratification Algorithm

In current European Society of Cardiology (ESC) guidelines, clinical scores represent the first step in the risk stratification of hemodynamically stable PE [2]. Because HESTIA, PESI, and sPESI do not account for biomarkers, these scores must be complemented by biological and imaging data to identify patients at risk of early deterioration.

This limitation underscores the growing interest in circulating biomarkers—both established and emerging—as tools to refine prognostic assessment beyond clinical variables alone. Accordingly, biomarkers play a pivotal role in distinguishing intermediate–low-risk from intermediate–high-risk PE and in guiding monitoring intensity and therapeutic escalation.

### 2.5. Additional Risk Stratification Scores Integrating Biomarkers

In addition to PESI/sPESI and HESTIA, several other validated prognostic scores have been developed to refine risk stratification in hemodynamically stable patients with acute pulmonary embolism, particularly within the heterogeneous intermediate-risk group.

The BOVA score is specifically designed to identify normotensive patients at increased risk of early complications [10]. It incorporates clinical variables (heart rate ≥110 bpm, systolic blood pressure 90–100 mmHg), imaging evidence of RV dysfunction, and elevated cardiac troponin levels. Patients are stratified into three stages associated with progressively higher risks of 30-day adverse outcomes, including PE-related mortality and hemodynamic collapse. This score illustrates the added prognostic value of combining clinical, imaging, and biomarker data in a single model.

Similarly, the FAST score (H-FABP, syncope, and tachycardia) integrates a biomarker reflecting early myocardial injury—heart-type fatty acid-binding protein (H-FABP)—with clinical parameters [11]. This approach enables early identification of normotensive patients at risk of deterioration, even in cases where conventional biomarkers such as troponins may still be negative.

These models highlight the potential of multimodal approaches combining clinical findings, imaging, and biomarkers. However, despite their validated prognostic performance, these scores are not currently incorporated into ESC guideline-recommended algorithms, which continue to rely on a stepwise strategy integrating clinical assessment, imaging, and biomarkers rather than a single composite score.

The aim of this review is to provide a practical and up-to-date overview of the role of different biomarker families in the prognostic assessment of pulmonary embolism.

## 3. Cardiac Biomarkers in the Prognostic Assessment of Pulmonary Embolism

Because clinical risk scores such as HESTIA, PESI, and sPESI rely exclusively on clinical variables and do not incorporate biological data, the assessment of circulating cardiac biomarkers constitutes a critical second step in the prognostic stratification of hemodynamically stable PE. These biomarkers reflect RV pressure overload, myocardial injury, or ventricular strain, which are central pathophysiological mechanisms driving early morbidity and mortality in acute PE.

According to ESC guidelines, cardiac biomarkers are primarily used to refine risk stratification in patients classified as intermediate risk based on clinical scores [2]. When combined with imaging evidence of RV dysfunction, biomarkers allow the distinction between intermediate–low-risk and intermediate–high-risk PE, thereby influencing decisions regarding monitoring intensity and potential escalation of therapy.

### 3.1. Cardiac Troponins

In acute PE, sudden obstruction of the pulmonary vasculature results in an abrupt increase in RV afterload, leading to RV dilatation, increased wall stress, and subendocardial ischemia. This myocardial injury is reflected by the release of cardiac troponins (troponin I and troponin T), which are sensitive and specific markers of myocardial damage. In the context of acute PE, troponin elevation reflects RV ischemia secondary to acute pressure overload rather than underlying coronary artery disease.

RV dysfunction is a major determinant of prognosis in acute PE and is associated with an increased risk of adverse short-term outcomes, including all-cause mortality, PE-related death, and hemodynamic deterioration, even in normotensive patients [12,13]. Although bedside echocardiography is a cornerstone of risk stratification by allowing early detection of RV dysfunction, its use may be limited by availability, operator dependency, and cost. In this setting, cardiac troponin measurement provides valuable and complementary prognostic information, particularly in hemodynamically stable patients.

Numerous studies have demonstrated a strong association between elevated troponin levels and poor outcomes in acute PE. Both conventional and high-sensitivity troponin assays have shown prognostic value, with elevated levels associated with a 2- to 5-fold increase in early mortality compared with normal values [12,13]. Elevated troponin concentrations have been consistently linked to RV dysfunction, as demonstrated by echocardiographic findings such as RV dilatation and increased pulmonary vascular obstruction on lung imaging. Moreover, high troponin T levels have been associated with adverse 30-day clinical outcomes and increased long-term mortality [12,13,14].

Importantly, a normal troponin level carries a high negative predictive value, identifying patients at low risk of early complications [15]. Compared with other prognostic markers, the predictive performance of troponins is similar to or slightly inferior to the simplified Pulmonary Embolism Severity Index (sPESI); however, their combined use improves risk stratification accuracy [16].

Consequently, current ESC guidelines recommend troponin assessment for prognostic evaluation in hemodynamically stable patients with acute PE and an sPESI ≥ 1, as part of a multimodal risk assessment strategy [2].

### 3.2. Natriuretic Peptides (BNP and NT-ProBNP)

Brain natriuretic peptide (BNP) is a neurohormone released by the cardiac ventricles in response to ventricular wall stress. BNP and its inactive fragment, N-terminal pro–B-type natriuretic peptide (NT-proBNP), are secreted following cleavage of pro-BNP and are released in response to RV pressure overload. In acute PE, elevated BNP and NT-proBNP levels reflect RV dilatation and dysfunction secondary to increased pulmonary vascular resistance [17].

BNP and NT-proBNP have been proposed, together with cardiac troponins, as useful biomarkers for identifying RV dysfunction and for predicting mortality and serious adverse events in patients with acute PE [14]. Multiple studies and meta-analyses have consistently shown that elevated levels of natriuretic peptides are associated with increased short-term mortality and a higher risk of clinical deterioration. In a large meta-analysis including 1132 unselected patients with acute PE, 51% had elevated BNP or NT-proBNP concentrations at admission, with an associated early mortality risk of 10% (95% CI 8.0–13%) and an unfavorable clinical outcome rate of 23% (95% CI 20–26%) [18].

From a physiological perspective, BNP plays a role in sodium homeostasis and blood pressure regulation, inducing systemic vasodilation and pulmonary artery dilation. NT-proBNP, although biologically inactive, is present in plasma at concentrations five- to ten-fold higher than BNP and has a longer half-life (approximately 2 h versus 20 min for BNP), making it particularly suitable for laboratory measurement. Both peptides are primarily eliminated by the kidneys.

Although natriuretic peptides have prognostic value, current ESC/ERS guidelines do not recommend their use alone to guide outpatient management decisions. In clinical practice, BNP and NT-proBNP are mainly used for their high negative predictive value, allowing the identification of a subgroup of low-risk PE patients who may be eligible for outpatient management. However, their specificity remains limited, as elevated levels may be influenced by advanced age, renal dysfunction, chronic heart failure, or atrial fibrillation [19]. Consequently, natriuretic peptide levels should be interpreted in conjunction with clinical findings and other prognostic markers to optimize risk stratification in patients with acute PE.

### 3.3. D-Dimers

D-dimers are degradation products of cross-linked fibrin and reflect both fibrin formation and fibrinolysis [20]. Under physiological conditions, a tightly regulated balance between coagulation and fibrinolysis ensures effective hemostasis while preventing unnecessary thrombus persistence. Once a clot is no longer required, tissue-type and urokinase-type plasminogen activators convert plasminogen into plasmin, initiating fibrinolysis. Plasmin-mediated degradation of fibrin results in the release of fibrin degradation products, including D-dimers. In acute PE, elevated D-dimer concentrations indicate the presence of intravascular thrombosis and ongoing clot resolution.

In contrast to cardiac biomarkers, which primarily reflect RV injury or strain, D-dimers are predominantly used for diagnostic purposes [21]. Owing to their very high sensitivity, D-dimer testing is a cornerstone of diagnostic algorithms for suspected PE in patients with a low or intermediate pretest clinical probability. Current international guidelines identify D-dimers as the only laboratory biomarker routinely incorporated into PE diagnostic pathways [22].

When D-dimer levels are below the appropriate threshold, the test demonstrates excellent diagnostic performance, with a sensitivity of approximately 96% and a negative predictive value (NPV) of 94% for PE [23]. In patients with D-dimer concentrations ≤ 0.5 µg/mL, the NPV for venous thromboembolism has been reported to approach 100% [24]. Accordingly, a normal D-dimer level reliably excludes deep vein thrombosis and PE in patients with a low clinical probability, thereby avoiding unnecessary imaging [25]. D-dimer testing is not used to confirm the diagnosis of PE but rather to safely exclude PE in patients with low or intermediate clinical probability, typically assessed using validated scores such as the Wells or Geneva score [26]. In this context, a negative D-dimer result allows clinicians to rule out PE without the need for imaging.

Beyond their diagnostic role, the prognostic implications of D-dimer levels have been increasingly explored. Elevated D-dimer concentrations are independently associated with an increased risk of PE, greater thrombotic burden, and higher short-term mortality [27]. However, unlike troponins or natriuretic peptides, D-dimers are not markers of RV dysfunction and are therefore not included in current ESC risk stratification algorithms for defining intermediate-risk categories.

Interpretation of D-dimer levels is particularly challenging in populations with chronically elevated baseline values, including elderly patients, pregnant women, patients with active cancer, and those with renal dysfunction. In these settings, diagnostic specificity is reduced when fixed cut-off values are used, leading to high rates of false-positive results [28].

To address this limitation, adjusted D-dimer thresholds have been developed and validated. Age-adjusted cut-offs improve diagnostic efficiency in older patients without compromising safety [22]. Similarly, combining D-dimer concentrations with clinical risk scores, such as the sPESI, appears to improve prognostic discrimination, supporting the concept of multivariable clinical–biological models rather than reliance on isolated biomarkers [27].

Physiological increases in D-dimer levels during pregnancy limit the utility of conventional thresholds. Nevertheless, when used within pregnancy-adapted diagnostic algorithms, D-dimer testing may help exclude PE in selected patients and reduce exposure to ionizing radiation. In patients with cancer, where D-dimer levels are frequently elevated, increasing the diagnostic threshold (e.g., to 700 µg/L) has been shown to safely increase the proportion of patients in whom PE can be excluded without imaging, supporting cancer-specific adaptations [28].

More recently, diagnostic strategies integrating D-dimer levels with clinical probability-adjusted algorithms, such as the YEARS algorithm, have been developed to improve diagnostic efficiency while safely reducing the need for imaging [29,30].

In summary, D-dimers differ fundamentally from cardiac and emerging biomarkers in PE. While they are central to diagnosis, their role in prognostic stratification remains adjunctive and indirect. D-dimers may contribute to integrated risk assessment when combined with clinical scores, imaging, and cardiac biomarkers, but they do not replace markers of RV dysfunction. This distinction highlights the complementary roles of biological markers across the diagnostic–prognostic continuum in acute pulmonary embolism. Table 4 summarizes the main biomarkers used in the assessment of pulmonary embolism, outlining their clinical significance and corresponding normal reference values.

### 3.4. Combined Use of Cardiac Biomarkers and Imaging

The prognostic value of cardiac biomarkers is maximized when combined with imaging evidence of RV dysfunction assessed by echocardiography or computed tomography pulmonary angiography. This multimodal strategy underpins the ESC-defined intermediate-risk categories (see Table 5):

Patients with intermediate–high-risk PE are at particular risk of early hemodynamic decompensation and should be monitored in a high-dependency or intensive care setting to allow prompt initiation of rescue reperfusion therapy if clinical complications occur.

### 3.5. Limitations of Conventional Cardiac Biomarkers

Despite their established role, conventional cardiac biomarkers have limitations. Elevations are not specific to PE, and optimal cut-off values may vary depending on the assay used and patient characteristics. Moreover, biomarkers are static measurements that may not fully capture dynamic changes in RV function over time. These limitations have driven interest in additional or emerging biomarkers that may improve risk stratification, particularly within the heterogeneous intermediate-risk population [31].

This provides a natural transition toward a following section on emerging biomarkers, such as copeptin, heart-type fatty acid-binding protein (H-FABP), growth differentiation factor-15 (GDF-15), and microRNAs.

## 4. Readily Available Biomarkers with Emerging Prognostic Applications in Pulmonary Embolism

Given the limitations of conventional cardiac biomarkers, considerable interest has focused on non-traditional prognostic biomarkers that may improve prognostic accuracy in acute PE, particularly among patients classified as intermediate risk. These biomarkers reflect complementary pathophysiological pathways, including neurohormonal activation, myocardial stress, inflammation, hypoxia, and cellular injury, and may allow earlier or more refined identification of patients at risk of clinical deterioration.

### 4.1. Copeptin

Copeptin (CT-proAVP), the C-terminal fragment of the arginine vasopressin (AVP) precursor pre-provasopressin, is released in equimolar amounts with AVP in response to hemodynamic stress and decreased cardiac output. Because of its greater stability and longer half-life compared with AVP, copeptin represents a reliable surrogate marker of endogenous vasopressin release and acute endogenous stress. Normal serum levels of CT-proAVP range from 1.70 to 11.25 pmol/L.

In acute PE, elevated copeptin levels have been consistently associated with increased short-term mortality, hemodynamic instability, and adverse clinical outcomes [32]. Several observational studies have demonstrated that copeptin provides independent prognostic information beyond established clinical risk scores and conventional biomarkers [33]. Importantly, copeptin may identify patients at high risk of early clinical deterioration even when cardiac troponin and natriuretic peptide levels are within the normal range, suggesting a potential role in the early detection of patients at risk of sudden decompensation [34].

In a monocentric study including 268 normotensive patients with acute PE, CT-proAVP levels ≥24 pmol/L were associated with a 5.4-fold increased risk of poor prognosis (95% CI 1.7–17.6) [35]. These findings were subsequently confirmed in 843 normotensive PE patients prospectively enrolled in three European cohorts, supporting the robustness of copeptin as a prognostic biomarker in this population [36].

Despite these promising results, the clinical use of copeptin remains limited by its lack of disease specificity and its restricted availability in routine practice. Consequently, copeptin is currently considered an emerging biomarker that may complement, rather than replace, established prognostic tools in the risk stratification of patients with acute PE.

### 4.2. Heart-Type Fatty Acid-Binding Protein (H-FABP)

Heart-type fatty acid-binding protein (H-FABP) is a small cytosolic myocardial protein (15 kDa) involved in intracellular fatty acid transport and metabolism. It is rapidly released into the circulation following myocardial ischemia, with detectable blood levels as early as 1 to 3 h after injury. Compared with cardiac troponins, H-FABP rises earlier and may therefore allow the detection of very early right RV myocardial damage in patients with acute PE [37].

In acute PE, elevated H-FABP levels have been consistently associated with increased short-term mortality and adverse clinical outcomes, even in hemodynamically stable patients. H-FABP appears to improve risk stratification within the intermediate-risk group and has been proposed as a biomarker capable of identifying patients at increased risk despite negative troponin results [38]. Consequently, its measurement has been recommended in conjunction with troponin rather than as a standalone biomarker.

A meta-analysis including 1,680 patients with acute PE demonstrated that H-FABP concentrations ≥6 ng/mL were strongly associated with unfavorable short-term clinical outcomes (odds ratio [OR] 17.7, 95% CI 6.0–51.9) and all-cause mortality (OR 32.9, 95% CI 8.8–123.2) [13]. These findings highlight the strong prognostic value of H-FABP in this setting.

Despite its promising performance, the routine clinical use of H-FABP remains limited by restricted assay availability and the lack of universally standardized cut-off values. Therefore, H-FABP is currently considered a complementary biomarker that may refine risk stratification in selected patients with acute PE.

### 4.3. Growth Differentiation Factor-15 (GDF-15)

Growth differentiation factor-15 (GDF-15) is a stress-responsive cytokine belonging to the transforming growth factor-β superfamily [39]. It is released in response to inflammation, oxidative stress, hypoxia, and tissue injury—all key mechanisms involved in acute PE.

Elevated GDF-15 concentrations have been associated with increased mortality and major adverse events in PE [40]. Unlike troponins or natriuretic peptides, GDF-15 integrates multiple pathophysiological processes, which may enhance prognostic performance. However, its low specificity and limited clinical availability currently confine its role to research settings [41].

### 4.4. Lactate

Serum lactate is a marker of tissue hypoperfusion and anaerobic metabolism, reflecting an imbalance between oxygen supply and demand. In acute PE, elevated lactate levels may be observed even in the absence of overt hypotension and can indicate occult circulatory failure, suggesting severe PE with manifest or imminent hemodynamic impairment [42].

Several studies have demonstrated that elevated arterial lactate levels (≥2 mmol/L) are associated with an increased risk of PE-related complications and mortality, both in unselected populations and in initially normotensive patients with acute PE [43]. This association appears particularly relevant among intermediate-risk patients, in whom lactate measurement may help identify those at higher risk of early clinical deterioration [44].

From a practical standpoint, lactate assessment is widely available, inexpensive, and rapidly obtainable, making it an attractive adjunctive biomarker for risk stratification in acute PE. However, its clinical interpretation requires caution, as lactate lacks specificity and may be influenced by concomitant conditions such as sepsis, hepatic dysfunction, or intense physical stress. Consequently, lactate levels should be interpreted in conjunction with clinical findings and other prognostic markers to refine risk assessment [42].

### 4.5. MicroRNAs (miRNAs)

MicroRNAs (miRNAs) are a class of small, endogenous, non-coding single-stranded RNA molecules, approximately 21 to 25 ribonucleotides in length, involved in post-transcriptional gene regulation. Their expression profiles are tissue-specific and vary significantly across disease states, particularly in thrombotic disorders and malignancies [45]. In PE, specific circulating miRNAs have been implicated in RV dysfunction, endothelial injury, inflammation, and thrombosis (Table 6).

Recent experimental and clinical studies have identified several circulating miRNAs with potential diagnostic and prognostic value in PE. Plasma levels of microRNA-134 have been shown to be significantly higher in patients with PE compared with controls, while circulating miR-28-3p has been proposed as a predictor for the diagnosis of acute PE [46]. More recently, circulating miR-1233 has emerged as a sensitive and specific biomarker for the early diagnosis of PE. These preliminary findings suggest that specific miRNA signatures may, in selected populations, outperform conventional biomarkers [46].

Beyond acute PE, other non-coding RNAs appear to play a role in long-term thromboembolic complications. Long non-coding RNAs (lncRNAs) have been implicated in the pathogenesis of chronic thromboembolic pulmonary hypertension (CTEPH), while complex regulatory networks involving miRNAs, lncRNAs, and target genes have been associated with disease progression [47]. In addition, circular RNAs (circRNAs), characterized by covalently closed continuous RNA loops, seem to be involved in pulmonary hypertension, and their targeted modulation may represent a future therapeutic approach [48].

Other emerging biomarkers related to inflammatory and vascular pathways have also been explored. Pentraxin 3 (PTX3), a plasma protein with pleiotropic effects, is increased in pulmonary hypertension and has been proposed as a potential biomarker for the early diagnosis of PE [49].

Despite their theoretical advantages, including high plasma stability and tissue specificity, the clinical implementation of miRNAs and other non-coding RNAs remains limited. Significant heterogeneity in detection methods, the absence of standardized assays, and the lack of large-scale validation studies currently preclude their routine use in clinical practice. miRNAs remain primarily research tools at this stage, and their role in prognostic stratification is not yet established for routine clinical practice. As such, these biomarkers remain primarily of research interest, with potential future applications in precision medicine for PE.

## 5. Inflammatory Biomarkers in Pulmonary Embolism

Inflammation plays a central role in the pathophysiology of venous thromboembolism and PE, contributing to endothelial dysfunction, thrombus propagation, and RVstress. In recent years, several readily available inflammatory biomarkers derived from routine laboratory tests have been investigated for their potential prognostic value in acute PE. Although none are currently incorporated into guideline-recommended risk stratification algorithms, these markers may provide complementary information when integrated into multivariable models.

### 5.1. Leukocyte-Derived Ratios: NLR, PLR, and LMR

The neutrophil-to-lymphocyte ratio (NLR), platelet-to-lymphocyte ratio (PLR), and lymphocyte-to-monocyte ratio (LMR) have emerged as surrogate markers of systemic inflammation and immune dysregulation. These ratios are easily calculated from complete blood counts and reflect the balance between inflammatory activation and immune response [50].

Among these indices, NLR has shown the most consistent association with clinical outcomes in acute PE. Elevated NLR values have been independently associated with increased in-hospital mortality, higher disease severity, and adverse short-term outcomes. NLR may therefore contribute to early risk stratification, particularly in settings where advanced biomarker testing is not readily available. PLR and LMR have also been associated with PE severity, although their prognostic performance appears less robust and more variable across studies [51].

### 5.2. Red Cell Distribution Width (RDW)

Red cell distribution width (RDW), a measure of erythrocyte size variability, has been increasingly recognized as a marker of chronic inflammation, oxidative stress, and impaired erythropoiesis. Elevated RDW levels reflect a pro-inflammatory milieu and have been associated with adverse outcomes across a range of cardiovascular and pulmonary diseases [52].

In acute PE, increased RDW has been consistently associated with higher mortality and worse clinical outcomes. Recent meta-analyses have confirmed RDW as an independent prognostic indicator in patients with PE. Given its widespread availability and low cost, RDW represents an attractive adjunctive biomarker for prognostic assessment, although its lack of specificity limits standalone clinical use [53].

### 5.3. C-Reactive Protein (CRP)

C-reactive protein (CRP) is an acute-phase protein synthesized by the liver in response to inflammatory cytokines. High-sensitivity CRP (hs-CRP) assays allow precise quantification of low-grade systemic inflammation.

In patients with acute PE, elevated hs-CRP levels have been correlated with disease severity, thrombotic burden, and the extent of pulmonary vascular obstruction. Studies comparing hs-CRP levels before and after thrombolytic therapy have shown a significant decrease following reperfusion, particularly in patients with extensive embolic involvement [54]. These findings suggest that hs-CRP may serve as a dynamic biomarker for monitoring treatment response and prognostic assessment, although it lacks specificity for thromboembolic disease.

### 5.4. Pro-Inflammatory Mediators: TNF-α and HMGB1

Tumor necrosis factor alpha (TNF-α) and high-mobility group box 1 (HMGB1) are key inflammatory mediators implicated in endothelial activation, vascular inflammation, and thrombus formation. While their roles have been extensively studied in inflammatory and respiratory diseases such as acute lung injury, asthma, chronic obstructive pulmonary disease, and pulmonary fibrosis, data in acute PE remain limited [55].

Experimental and translational studies suggest that TNF-α promotes endothelial dysfunction, vascular inflammation, and thrombogenesis, and may contribute to pulmonary vascular remodeling and the development of chronic thromboembolic pulmonary hypertension. Similarly, HMGB1 has been implicated in venous thrombosis formation and propagation in animal models. However, clinical evidence supporting their prognostic utility in acute PE is currently insufficient, and their use remains confined to research settings [56].

#### Summary of Inflammatory Biomarkers in Pulmonary Embolism

Inflammatory biomarkers reflect complementary biological pathways involved in PE but currently lack sufficient specificity and validation to guide individual management decisions. Their greatest potential lies in multimarker approaches, where they may enhance prognostic stratification when combined with clinical scores, imaging findings, and established cardiac biomarkers. A detailed summary of inflammatory biomarkers and their clinical relevance in pulmonary embolism is presented in Table 7.

## 6. Perspectives and Future Directions

Plasma biomarkers play a central role in the diagnosis, risk stratification, and long-term management of PE. Although not cardiac-specific, D-dimer remains essential for the rapid exclusion of PE in patients with a low or intermediate clinical probability. In contrast, cardiac biomarkers provide key prognostic information by reflecting RV injury and hemodynamic stress, which are major determinants of outcome in acute PE.

Among these, cardiac troponins and B-type natriuretic peptide (BNP) or its N-terminal fragment (NT-proBNP) are the most widely used and best validated. Troponin elevation is consistently associated with increased short-term mortality, hemodynamic deterioration, and acute heart failure in severe PE. BNP and NT-proBNP reflect RV pressure overload and dysfunction, a hallmark of severe and intermediate-risk PE. Accordingly, cardiac biomarkers are now integrated into PE risk stratification algorithms, in combination with clinical scores and imaging findings. Intermediate-risk PE is defined by evidence of RV dilatation on imaging (RV/LV ratio >1) together with elevated cardiac troponin levels, guiding closer monitoring and therapeutic escalation. The association between markers of RV dysfunction and short-term mortality is detailed in Table 8.

Beyond conventional biomarkers, several emerging markers—including copeptin, lactate, creatinine elevation, heart-type fatty acid-binding protein (H-FABP), inflammatory and hematologic indices (NLR, PLR, LMR, RDW), and cytokines such as TNF-α and HMGB1—have shown potential prognostic value. These biomarkers reflect complementary pathophysiological pathways, including neurohormonal activation, tissue hypoperfusion, inflammation, and early myocardial injury, and may further refine risk stratification in selected patients.

Biomarkers may also contribute to therapeutic decision-making. In intermediate–high-risk PE, half-dose thrombolysis (fixed alteplase regimen, 50 mg over 2 h) has been associated with improved short-term respiratory outcomes and reduced long-term pulmonary hypertension, as demonstrated in the randomized MOPETT trial [59]. The MOPETT trial evaluated the use of reduced-dose thrombolysis in patients with so-called “moderate” pulmonary embolism, defined by a high thrombotic burden rather than by contemporary ESC risk stratification criteria. As such, this population does not strictly correspond to the current intermediate–high-risk category. Although biomarkers are not currently used in isolation to guide fibrinolytic therapy, elevated levels of troponin, BNP, or copeptin—reflecting significant RV strain—may support early, more aggressive treatment strategies in carefully selected patients to prevent hemodynamic deterioration and long-term sequelae.

Looking forward, the role of biomarkers in PE management is expected to expand with advances in omics technologies, including proteomics, transcriptomics, and genomics. Novel biomarkers, particularly circulating microRNAs, show promise for earlier detection of PE-related cardiac injury, improved prognostic assessment, and identification of subclinical or atypical disease presentations. Integration of biomarker profiling with advanced imaging modalities, such as cardiac MRI and three-dimensional echocardiography, may enable more precise and dynamic evaluation of RV function and pulmonary vascular burden.

Finally, biomarkers may play an increasingly important role in long-term follow-up and personalized care. D-dimer remains the most validated biomarker for guiding anticoagulation duration after venous thromboembolism, particularly in patients with unprovoked events. Emerging evidence suggests that microRNAs and other molecular markers may also contribute to long-term monitoring and could even represent future therapeutic targets in chronic post-embolic pulmonary hypertension. Together, these developments highlight the growing potential of biomarkers to support precision medicine across the entire spectrum of PE care.

**Figure 1:**
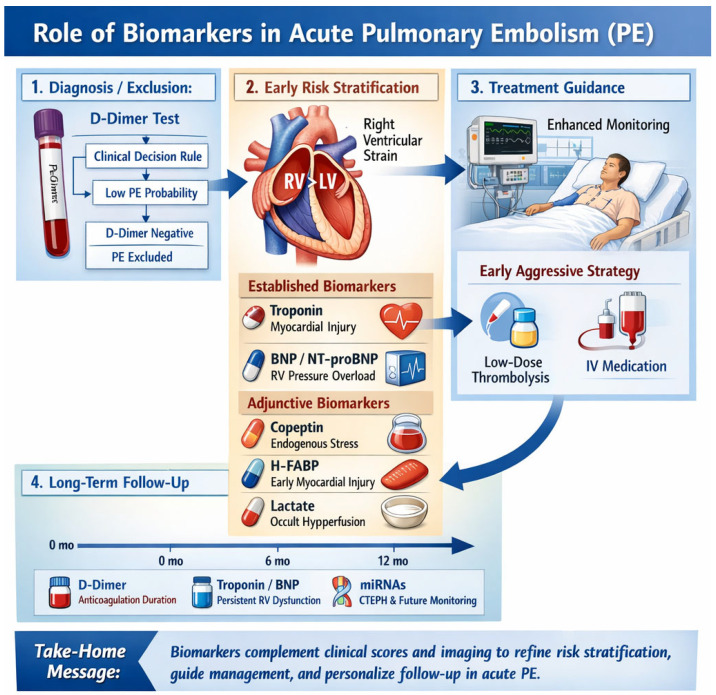
Role of biomarkers in acute pulmonary embolism.

The overall role of biomarkers in the diagnosis, risk stratification, treatment guidance, and long-term follow-up of acute PE is illustrated in Figure 1, emphasizing how they complement clinical probability assessment, imaging evaluation of RV dysfunction, and therapeutic decision-making throughout the continuum of care.

The development of integrated risk scores combining clinical variables, imaging findings, and biomarkers remains an area of active research, with models such as BOVA and FAST illustrating the potential of this approach. However, current clinical practice continues to favor stepwise multimodal risk stratification.

## Figures and Tables

**Table 1:** HESTIA Criteria for Outpatient Management of Pulmonary Embolism.

Criterion	Description
Hemodynamic instability	Systolic blood pressure < 100 mmHg
Need for thrombolysis or embolectomy	Current or anticipated
Active bleeding	Or high risk of bleeding
Oxygen requirement	To maintain SpO_2_ > 90%
Severe pain	Requiring intravenous analgesia
Recent major bleeding	Within the previous 4 weeks
Thrombocytopenia	Platelet count < 75 × 10^9^/L
Severe renal failure	Creatinine clearance < 30 mL/min
Severe liver disease	With coagulopathy
Pregnancy	Confirmed or suspected
Concomitant medical condition	Requiring hospital admission
Inadequate home or social setting	Inability to ensure safe outpatient care

**Table 2:** Simplified Pulmonary Embolism Severity Index (sPESI).

Variable	Criterion	Score
Age	>80 years	+1
History of cancer	Active or previous	+1
Chronic cardiopulmonary disease	Heart failure or chronic lung disease	+1
Heart rate	≥110 beats/min	+1
Systolic blood pressure	<100 mmHg	+1
Arterial oxygen saturation	<90%	+1
Score interpretation:		
sPESI Score	Risk Category	Estimated 30-day Mortality
0	Low risk	~1%
≥1	Higher risk	8–10%

**Table 3:** Index Comparison and Clinical Use.

Feature	HESTIA	sPESI
Purpose	Outpatient eligibility	Mortality risk prediction
Type	Binary checklist	Numerical score
Includes social factors	Yes	No
Predicts 30-day mortality	No	Yes
Widely used in guidelines	Yes	Yes

**Table 4:** Summary of Biomarkers Used in Pulmonary Embolism Assessment.

Biomarker	Clinical Significance in Pulmonary Embolism	Normal Values
D-dimer	Useful for excluding pulmonary embolism in patients with low or intermediate clinical probability. Lacks specificity and may be elevated in many conditions (e.g., infection, cancer, pregnancy, postoperative state, advanced age).	<500 ng/mL (or age-adjusted threshold: age × 10 ng/mL for patients > 50 years)
High-sensitivity troponin T (hs-TnT)	Marker of myocardial injury secondary to acute right ventricular pressure overload. Used for risk stratification in combination with imaging: RV dilatation + elevated hs-TnT identifies intermediate–high and high-risk PE.	≤14 pg/mL (<75 years) ≤45 pg/mL (≥75 years)
BNP/NT-proBNP	Marker of right ventricular strain and pressure overload. Elevated levels are associated with worse prognosis and help identify intermediate-risk PE.	BNP < 100 pg/mL NT-proBNP < 125 pg/mL

**Table 5:** ESC Risk Stratification in Hemodynamically Stable Pulmonary Embolism.

Risk Category	Clinical Score (PESI/sPESI)	RV Dysfunction	Cardiac Biomarkers	Management Implications
Low risk	PESI I–II or sPESI = 0	Absent	Normal	Early discharge/outpatient treatment
Intermediate–low risk	PESI ≥ III or sPESI ≥ 1	Present or absent	Elevated or normal	Anticoagulation, standard monitoring
Intermediate–high risk	PESI ≥ III or sPESI ≥ 1	Present	Elevated	Anticoagulation, close monitoring, consider rescue reperfusion
High risk	—	—	—	Immediate reperfusion therapy

**Table 6:** Repurposed Biomarkers in Pulmonary Embolism Prognosis.

Biomarker	Pathophysiological Pathway	Prognostic Value	Main Limitations
Copeptin	Neurohormonal stress	Early mortality, shock	Low specificity, limited availability
H-FABP	Early myocardial injury	Short-term mortality	Assay availability, cut-offs
GDF-15	Inflammation, hypoxia	Mortality, adverse events	Low specificity
Lactate	Tissue hypoperfusion	Mortality, deterioration	Non-specific
miRNAs	Molecular regulation	Severity, RV dysfunction	Lack of standardization

**Table 7:** Overview of inflammatory biomarkers in pulmonary embolism.

Biomarker	Pathophysiological Role	Prognostic Association	Main Limitations
NLR	Systemic inflammation, immune imbalance	In-hospital mortality, severity	Non-specific
PLR	Platelet activation, inflammation	Severity	Variable evidence
LMR	Immune regulation	Severity	Limited validation
RDW	Chronic inflammation, oxidative stress	Mortality	Low specificity
hs-CRP	Acute-phase inflammation	Severity, treatment response	Non-specific
TNF-α	Endothelial inflammation, thrombosis	Experimental evidence	Limited clinical data
HMGB1	Thrombus formation, inflammation	Preclinical evidence	Research only

NLR: neutrophil-to-lymphocyte ratio; PLR: platelet-to-lymphocyte ratio; LMR: lymphocyte-to-monocyte ratio; RDW: red cell distribution width; hs-CRP: high-sensitivity C-reactive protein; TNF-α: tumor necrosis factor alpha; HMGB1: high-mobility group box 1.

**Table 8:** Prognostic value of markers of right ventricular dysfunction for short-term mortality (from [13,57,58]).

Marker	Sensitivity (95% CI)	Specificity (95% CI)	PLR (95% CI)	NLR (95% CI)
Troponin	0.66 (0.61 to 0.70)	0.66 (0.65 to 0.67)	2.13 (1.84 to 2.47)	0.51 (0.40 to 0.60)
BNP	0.88 (0.65 to 0.96)	0.70 (0.64 to 0.75)	2.13 (1.84 to 2.47)	0.51 (0.40 to 0.60)
NT-proBNP	0.93 (0.14 to 1.00)	0.58 (0.14 to 0.92)	2.93 (2.28 to 3.77)	0.17 (0.05 to 0.58)
RVD US	0.70 (0.46 to 0.86)	0.57 (0.47 to 0.66)	1.48 (1.05 to 2.08)	0.82 (0.65 to 1.03)
RVD CT	0.65 (0.35 to 0.85)	0.56 (0.39 to 0.71)	1.63 (1.27 to 2.08)	0.53 (0.31 to 0.89)

CI: confidence interval; PLR: positive likelihood ratio; NLR: negative likelihood ratio; BNP: brain natriuretic peptide; NT-proBNP: N-terminal brain natriuretic peptide; RVD: right ventricular dysfunction; US: ultrasonography; CT: computer tomography.

## Data Availability

No new data were created or analyzed in this study.
